# The Autoimmune Gender Gap: A Rare Case of a Male Patient With Overlapping Autoimmune Hepatitis and Primary Biliary Cholangitis

**DOI:** 10.7759/cureus.63312

**Published:** 2024-06-27

**Authors:** Katherine Chwa, Sammy Aung, Armando Reyes Yparraguirre, Connor Wayman, Omar Canaday

**Affiliations:** 1 Internal Medicine, University of Nevada Reno School of Medicine, Reno, USA

**Keywords:** pbc-aih overlap syndrome, male overlap syndrome, primary biliary cholangitis, autoimmune hepatitis, overlap syndrome

## Abstract

Autoimmune hepatitis (AIH) is a condition resulting in chronic, inflammatory changes to the liver. Primary biliary cholangitis (PBC) is an autoimmune condition that destroys intrahepatic bile ducts. Overlap syndrome with concomitant AIH and PBC comprises a rare subgroup of patients with immune-mediated liver disease, with incidence rates of male patients being exceedingly uncommon in a predominantly female patient population. Our case report investigates a rare case of a 41-year-old male patient diagnosed with overlapping AIH and PBC. He initially presented with symptoms of fatigue, pruritus, and episodes of Raynaud’s phenomenon, in addition to findings of persistently elevated liver enzymes despite lifestyle modifications. He had no past medical history, no history of alcohol use disorder, and no family medical history of chronic liver disease. Imaging did not reveal evidence of cirrhosis. Further diagnostic workup was significant for elevated immunologic markers for antinuclear antibodies (ANA) with positive centromere and cytoplasmic patterns, antimitochondrial antibodies (AMA) with F-actin antibodies, anti-smooth muscle antibodies (ASMA), and cytoplasmic antinuclear cytoplasmic antibodies (ANCA C). Liver biopsy showed prominent plasma cells and rare granulomas, consistent with the diagnosis of AIH with a component of PBC, respectively. He was started on ursodeoxycholic acid (UDCA), demonstrating a near-complete clinical response with resolution of symptoms and normalization of liver enzymes.

Studies investigating the low incidence of male patients with overlap syndrome are limited, as current research is overwhelmingly based on studies with predominantly female subjects. However, most studies generally recommend treatment with both UDCA and corticosteroids to reduce symptoms and biochemical markers. Our case report highlights a rare case of a male patient documenting excellent biochemical and clinical responses to monotherapy with UDCA. A possible theory is that our patient’s early treatment (prior to advanced disease progression) is associated with his near-complete biochemical response and symptomatic resolution on UDCA alone. Further research is needed to fully understand the clinical course and long-term prognosis of male patients with overlap syndrome. Our patient remains in life-long follow-up to monitor if or when he requires treatment with corticosteroids in addition to current monotherapy with UDCA.​

## Introduction

Autoimmune hepatitis (AIH) is a condition resulting in chronic, inflammatory changes to the liver. Primary biliary cholangitis (PBC) is an autoimmune condition that destroys intrahepatic bile ducts. Overlap syndrome with concomitant AIH and PBC comprises a rare subgroup of patients with immune-mediated liver disease, with the incidence rates of male patients being exceedingly uncommon in a predominantly female patient population [1,2]. Studies are somewhat limited given low disease incidence and lack of standardized criteria. However, current guidelines suggest patients with seropositive overlap syndrome require treatment with combination therapy of both ursodeoxycholic acid (UDCA) and corticosteroids to achieve clinical response. This case report highlights a rare diagnosis of a male patient with biopsy-proven, seropositive, overlapping AIH and PBC demonstrating a near-complete clinical response to monotherapy with UDCA.​

## Case presentation

**Figure 1:**
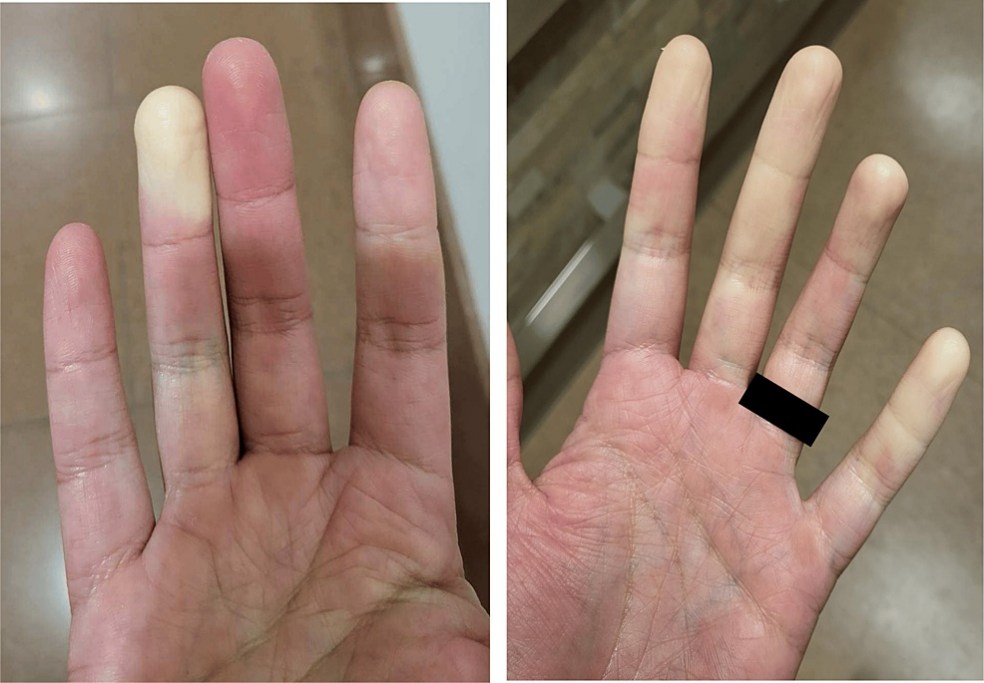
Clinical evidence during the onset of an episode of the patient’s Raynaud’s phenomenon.

A 41-year-old male with no past medical history presented with findings of elevated liver enzymes, ongoing for the past seven years, with concurrent symptoms of fatigue, pruritus, and episodes of Raynaud’s phenomenon (Figure 1). Their family history was negative for chronic liver disease. The patient stated he drank alcohol socially and denied any tobacco or illicit drug use. The physical exam was unremarkable. He was instructed by his primary care provider to implement lifestyle changes, adopt a healthy diet, and complete abstinence from alcohol. Despite these lifestyle changes, liver enzymes remained persistently elevated.​

**Figure 2:**
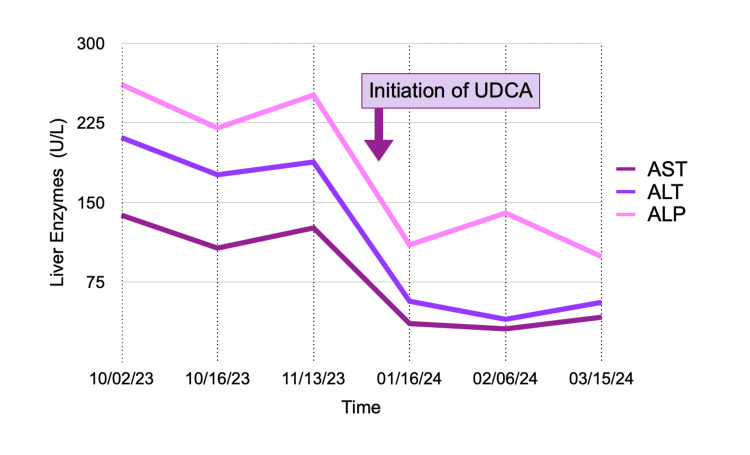
Trend of liver enzymes after treatment with UDCA. UDCA, ursodeoxycholic acid

Initial values of liver enzymes are as follows: aspartate transaminase (AST): 138, alanine transaminase (ALT): 211, and alkaline phosphatase (ALP): 261. Repeat laboratory testing after implementation of lifestyle changes showed minimal improvement: AST: 126, ALT: 188, and ALP: 251 (Figure 2). The lipid panel was within normal limits aside from findings of elevated HDL. ​

Ultrasound of the abdomen showed hepatomegaly. Transient elastography (FibroScan) showed minimal fatty infiltration and no fibrosis. Magnetic resonance cholangiopancreatography (MRCP) showed evidence of tiny liver cysts but was otherwise unremarkable.​

**Figure 3:**
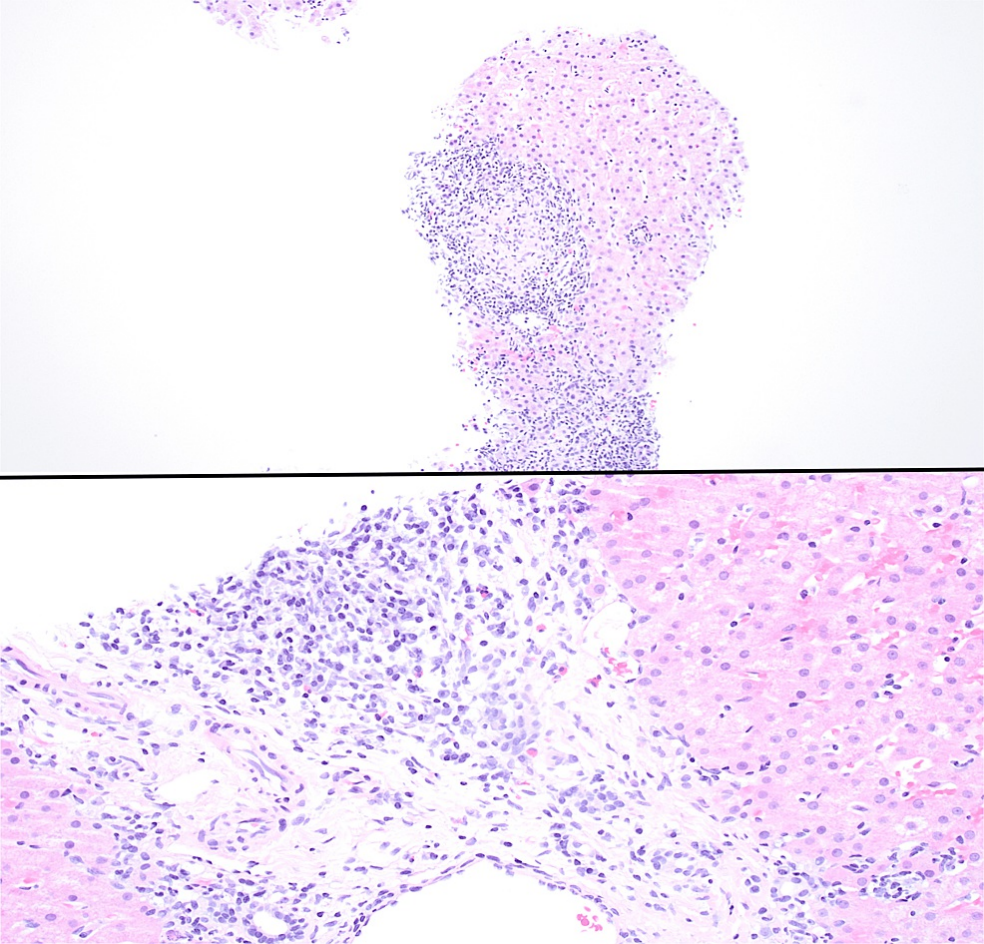
Images from the patient’s liver biopsy revealing a florid duct lesion with granulomatous destruction consistent with PBC (top) and plasma cell infiltrates consistent with AIH (bottom). PBC, primary biliary cholangitis; AIH, autoimmune hepatitis

Given the persistent elevation in liver enzymes and significant clinical history, further diagnostic workup was completed to assess for underlying autoimmune liver disease. Results revealed elevated immunologic markers for antinuclear antibodies (ANA) with positive centromere and cytoplasmic patterns, antimitochondrial antibodies (AMA) with F-actin antibodies, anti-smooth muscle antibodies (ASMA), and cytoplasmic antinuclear cytoplasmic antibodies (ANCA C). Laboratory workup was negative for elevated iron and percent saturation, hepatitis viruses A-B-C, alpha-1-antitrypsin, ceruloplasmin, anti-double stranded deoxyribonucleic acid antibodies (anti-dsDNA), anti-histidyl-tRNA synthetase antibodies (anti-Jo-1), anti-topoisomerase I antibodies (anti-Scl-70), anti-Smith antibodies, and anti-Sjogren’s syndrome A and B antibodies. Liver biopsy showed prominent plasma cells and rare granulomas, consistent with the diagnosis of AIH with a component of PBC, respectively (Figure 3). ​

Treatment was initiated in December of 2023 with UDCA 750 mg by mouth in the morning, followed by 500 mg by mouth in the evening. The patient reported excellent symptomatic response to therapy with near-resolution of his fatigue and pruritis. He tolerated treatment without any adverse effects. Repeat laboratory testing showed improved liver biochemistry with AST of 36, ALT of 57, and ALP of 110 (Figure 2), and additional treatment with corticosteroids was deferred. The patient remains in life-long follow-up to monitor treatment response; until now, liver enzymes have remained stable on monotherapy with UDCA. ​

## Discussion

While several factors (including sex hormones and epigenetics) play a role in AIH and PBC, the exact mechanism behind the low incidence in male patients remains largely unclear. Current data is overwhelmingly based on studies with female subjects, and even fewer studies have been published studying male subjects with overlap syndrome. Current data supports that both disease processes are secondary to abnormalities in T-cell function resulting in autoimmune destruction of liver architecture, with AIH leading to injury of liver parenchyma and PBC leading to injury of small bile ducts. ​

In AIH, high estrogen levels have been associated with increased cytokine levels, hypothetically resulting in increased destruction of liver cells by activation of cytotoxic T-cells [3]. It should be noted that this hypothesis was concluded based on a study conducted on mice. Although the data is limited, this may explain the prevalence of exacerbations of AIH during pregnancy [4]. Studies have also linked the development of PBC to incomplete X chromosome inactivation in females [5,6]. One study explored the genetics of inactive X chromosomes in discordant and concordant female monozygotic twins with PBC, with findings of two genes (CLIC2 and PIN4) that exhibited consistent down-regulation in subjects with PBC [7,8]. While this does suggest an association, it is important to note that neither gene was significantly correlated to transcription levels. ​

In our case, the patient responded well to monotherapy with UDCA, which contrasts with the usual treatment in patients with overlap syndrome; generally, combination therapy with UDCA and corticosteroids is recommended to reduce symptoms and biochemical markers. While most meta-analyses support the use of combination therapy for a complete biochemical response, one case series revealed that patients without biopsy findings of severe interface hepatitis achieved successful biochemical improvement on monotherapy with UDCA [9-11]. Notably, of the 88 patients enrolled in this study, 74 were female and 14 were male. Our patient did not have biopsy findings of severe interface hepatitis, which likely indicated his success with UDCA monotherapy. ​

## Conclusions

While more research is needed to establish more definitive conclusions on AIH, PBC, and overlap syndrome, studies have yet to be published exploring the occurrence of either disease process in male subjects. Our case report highlights a rare case of a male patient documenting excellent biochemical and clinical responses to monotherapy with UDCA. A possible theory is that our patient’s early treatment (prior to advanced disease progression) is associated with his near-complete biochemical response and symptomatic resolution on UDCA alone. Further research is needed to fully understand the clinical course and long-term prognosis of male patients with overlap syndrome. Our patient remains in life-long follow-up to monitor if or when he requires treatment with corticosteroids in addition to current monotherapy with UDCA.​
